# Infectious Pyomyositis With Intramuscular Abscess in a Healthy Adult

**DOI:** 10.7759/cureus.19676

**Published:** 2021-11-17

**Authors:** Francisco Dá Mesquita Faustino, Frederico Batista

**Affiliations:** 1 Intensive Care Unit, Hospital Prof. Doutor Fernando Fonseca, Amadora, PRT; 2 Department of Internal Medicine IV, Hospital Prof. Doutor Fernando Fonseca, Amadora, PRT

**Keywords:** pyomyositis, infectious disease, pathology, internal medicine, febrile syndrome, intramuscular abscess

## Abstract

Pyomyositis is an uncommon bacterial infection of the skeletal muscle, with most cases presenting with an intramuscular abscess. Although it is more frequent in tropical regions, it can also occur in temperate regions, essentially in adult males with comorbidities. We report a case of an adult male with an intramuscular abscess and demonstrate the importance of meticulous clinical examination and imaging examinations in obtaining a diagnosis for pathologies with nonspecific clinical manifestations.

## Introduction

Pyomyositis is an uncommon bacterial infection of the skeletal muscle frequently characterized by the formation of an intramuscular abscess. While tropical pyomyositis mainly affects children between two and five years and adults between 20 and 45 years, the temperate climate variant occurs mainly in adult males with comorbidities. The conditions associated with the development of pyomyositis include immunodeficiency states (human immunodeficiency virus infection, diabetes mellitus, systemic lupus erythematosus, neoplasia, chronic liver disease, chronic kidney disease, or immunosuppressive therapy), history of trauma, use of intravenous drugs, malnutrition, or a concomitant infection [1]. The most frequent etiological factor is *Staphylococcus aureus* [2]. Some cases of polymicrobial polymyositis have also been described [3]. Clinical manifestations include fever and pain in the muscles involved.

Natural history involves three phases. The first phase is called the invasive phase, which lasts for 10-21 days and is characterized by fever, anorexia, and diffuse and disabling muscle pain; only 2% of patients seek healthcare. The second phase is the purulent or suppurative phase, which includes more intense pain; in this phase, intramuscular pus collection develops, the affected muscle becomes painful on palpation, and the surrounding skin may be erythematous. The final phase may be characterized by the extension of the infection to the adjacent bone surface, with accompanying osteomyelitis or even progression to septic shock [4]. Pyomyositis seems to be secondary to septic embolization and can also occur due to muscle contusion or muscle necrosis after intense physical exertion [5]. Although the incidence of pyomyositis is rare, the mortality rate is significant and is estimated to be around 10% [6].

## Case presentation

We report a case of a 62-year-old Caucasian male with a prior history of prostate adenocarcinoma treated with radical retropubic prostatectomy, hypertension, and dyslipidemia who came to the emergency department of the Hospital Prof. Doutor Fernando Fonseca in Lisbon after several visits to other emergency departments. He complained of fever with chills for nine days, asthenia, anorexia without weight loss, and neck pain. He also reported an episode of a recent fall with left humeral and tibiotarsal trauma. On the examination, he had tenderness and pain on the left trapezius on the passive mobilization of the ipsilateral ankle. Laboratory analysis revealed leukocytosis (38,000/µL) with neutrophilia (34,960/µL), C-reactive protein of 42 mg/dL, and hypoxemia in blood gas (PaO2: 58 mmHg). A computed tomography (CT) scan of the left lower limb ruled out any fractures.

**Figure 1:**
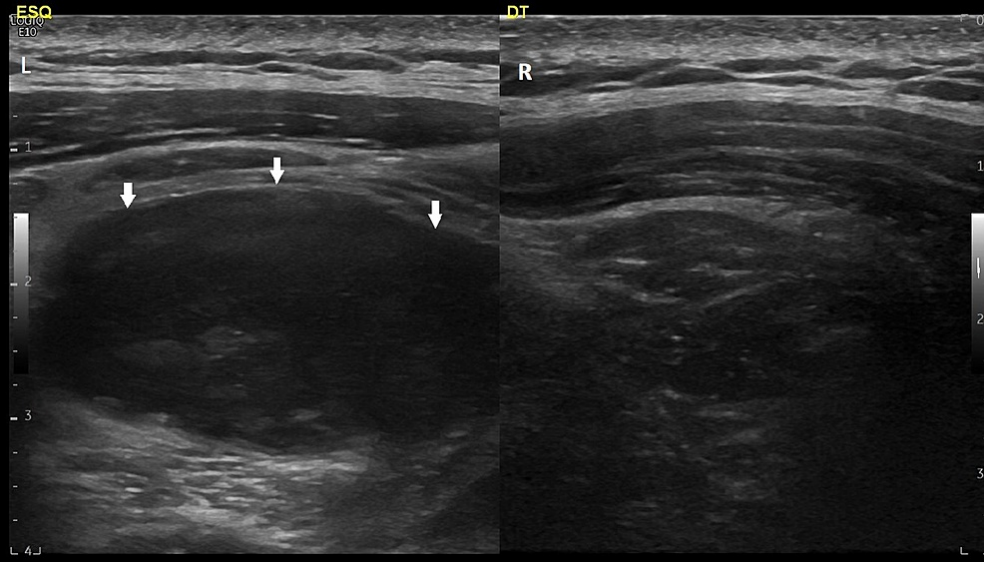
Heterogeneous collection (arrows) seen in ultrasound From: Ana Sofia Costa, Department of Radiology, Hospital Prof. Doutor Fernando Fonseca

We admitted the patient to investigate the etiology of his fever. Pulmonary and cardiac infectious foci were excluded based on the imaging results. During hospitalization, the patient remained febrile, with approximately two peaks of fever per day. The cervical pain got worse with the development of left cervical swelling with cervical asymmetry. A cervical ultrasound was requested (Figure 1), which revealed a heterogeneous collection in the thickness of the left trapezius muscle. This collection extended posteriorly and inferiorly along the muscular body (dorsal region) to 10 cm above the ipsilateral scapular angle, with an estimated maximum diameter of 30 mm.

**Figure 2:**
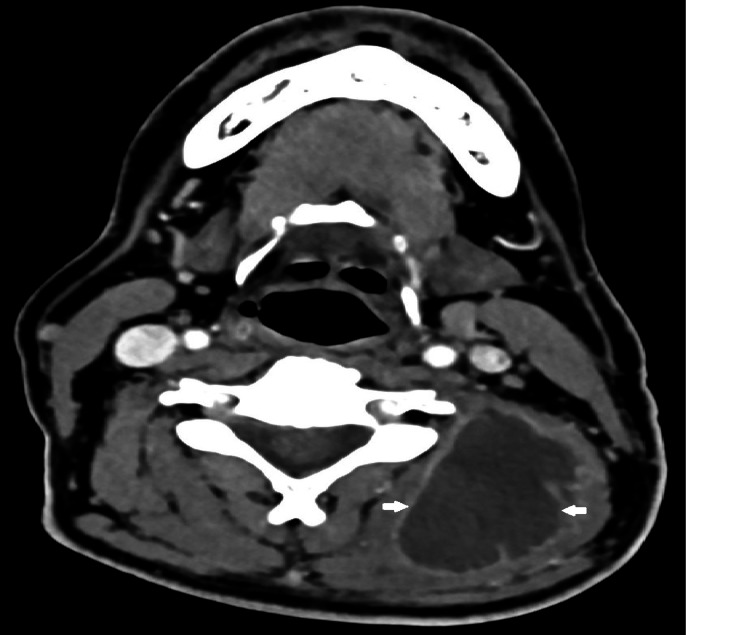
Cross view of the abscess at C3 level (arrows) From: Ana Sofia Costa, Department of Radiology, Hospital Prof. Doutor Fernando Fonseca

**Figure 3:**
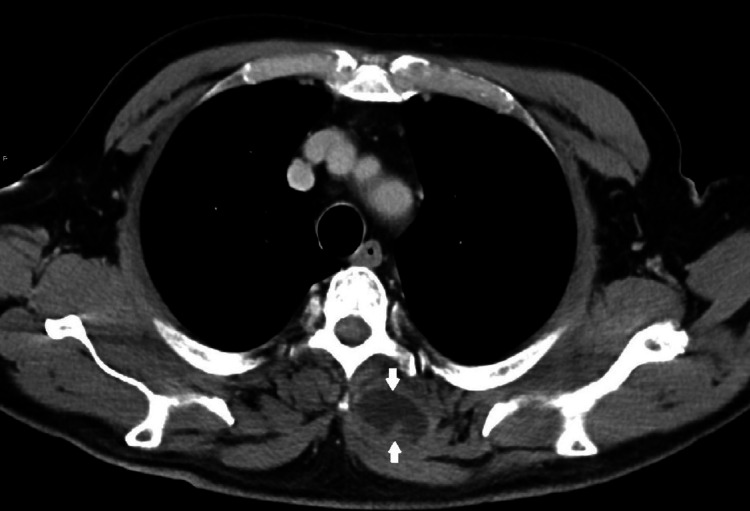
Cross view of the abscess at T5 level (arrows) From: Ana Sofia Costa, Department of Radiology, Hospital Prof. Doutor Fernando Fonseca

**Figure 4:**
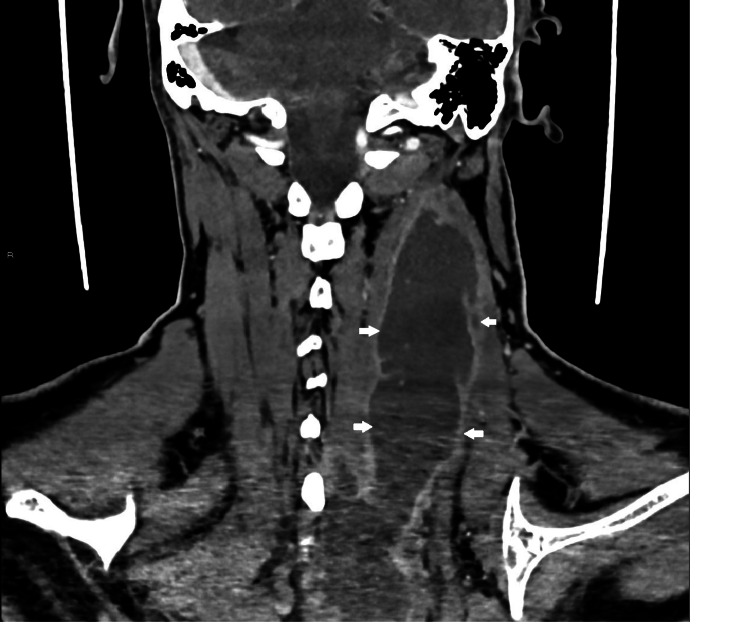
Coronal view of the abscess (arrows) From: Ana Sofia Costa, Department of Radiology, Hospital Prof. Doutor Fernando Fonseca

**Figure 5:**
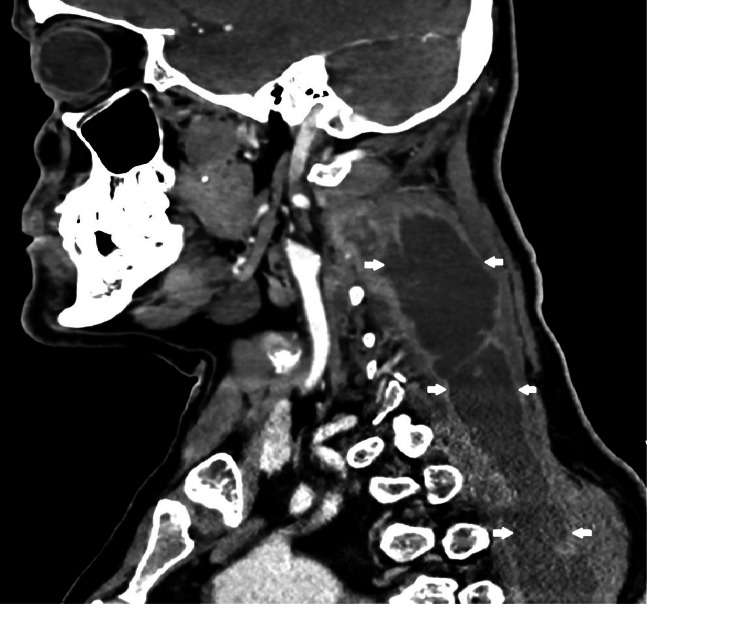
Sagittal view of the abscess (arrows) From: Ana Sofia Costa, Department of Radiology, Hospital Prof. Doutor Fernando Fonseca

**Figure 6:**
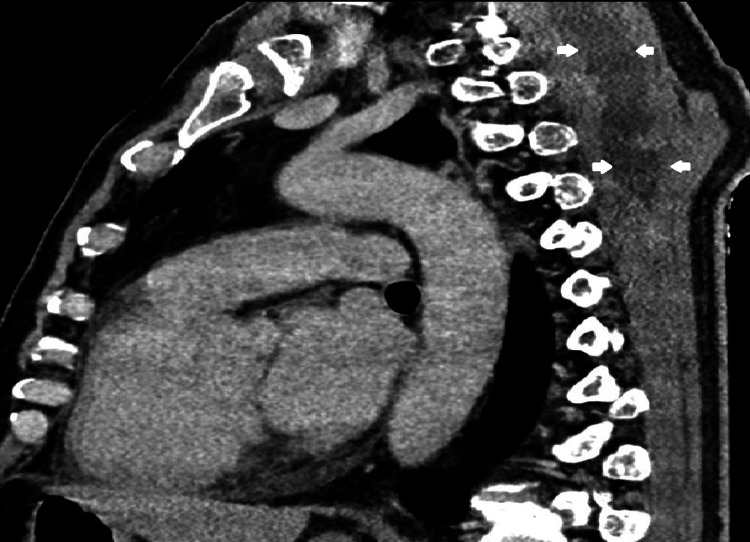
Sagittal view of the abscess at T5 level (arrows) From: Ana Sofia Costa, Department of Radiology, Hospital Prof. Doutor Fernando Fonseca

On the third day of hospitalization, a puncture with ultrasound guidance was performed, extracting purulent content for cultural examination. On the fourth day after admission, neck and chest CT were requested to characterize the abscess and showed a liquid collection with contrast uptake from the cervical region to the dorsal region at maximum extension in the axial plane of 54 × 36 mm and in the longitudinal plane of 20 cm (Figures 2-6). Therefore, we assumed trapezius pyomyositis with extensive paraspinal abscess from C3 to T5 and started empirical antibiotic therapy with ceftriaxone.

The patient subsequently underwent abscess drainage under general anesthesia with cytological, microbiological, and pathological analysis. The anatomopathological report revealed muscle and adipose tissue with degenerative aspects with an intense abscessed acute inflammatory process and necrotic fragments, without evidence of malignant neoplasia. Colonies of cocci in chains were observed. However, the abscess and blood cultures were negative, and the etiological agent could not be isolated.

We searched for predisposing conditions for infection and associated conditions with pyomyositis. However, only protein electrophoresis revealed an alteration in the gamma globulin range, but not a monoclonal component in the immunofixation. The 24-hour urinalysis showed no proteinuria or Bence Jones proteinuria, and full-body radiography ruled out lytic lesions. The patient was discharged on the 18th day with the indication to complete antibiotic therapy for four weeks and to be reassessed at internal medicine and general surgery appointments.

## Discussion

Pyomyositis is an uncommon infection with nonspecific clinical manifestations. Therefore, the diagnosis is delayed in most cases, with the diagnosis being established, on average, about 10 days from the onset of symptoms [7]. In the literature, more than half of the cases were diagnosed in the suppurative phase [4], despite the widespread use of imaging tests that would have enabled an earlier diagnosis. Similar to clinical manifestations, the diagnostic workup is not very specific, making the diagnosis of pyomyositis challenging and difficult to distinguish from other pathologies, such as muscle contusion, hematoma, cellulitis, septic arthritis, deep vein thrombosis, and osteomyelitis, among others.

In this case, in addition to the increase in inflammatory parameters, creatinine kinase levels were normal as in a significant percentage of cases in the literature, suggesting that the abscess initially develops among the muscle fibers without causing rhabdomyolysis [8]. Imaging examinations help to establish the diagnosis, particularly the ultrasound. Despite its poor sensitivity (especially in the early stages of the disease), it is an imaging technique without adverse effects, widely available, and helpful in viewing heterogeneous liquid collections. CT may be used to better characterize the lesion, as it possesses a higher spatial resolution. Other tests, such as magnetic resonance imaging or bone scintigraphy, are less frequently available in clinical practice and remain helpful in just a minority of cases in order to exclude other pathologies, such as septic arthritis or osteomyelitis.

Regarding treatment, when the disease is identified early (invasive phase), antibiotic therapy may be sufficient. However, in more advanced stages, such as in this case, drainage of the abscess is necessary to control the infectious focus. In immunocompetent individuals, antibiotic therapy should be directed against *Staphylococcus* and β-hemolytic streptococcus. In immunocompromised individuals, broader-spectrum antibiotic regimens should be considered [4].

In this patient, malnutrition, a concomitant infection, use of intravenous drugs, and immunodeficiency states, such as human immunodeficiency virus infection, diabetes mellitus, systemic lupus erythematosus, neoplasia, chronic liver disease, chronic kidney disease, or immunosuppressive therapy, were excluded. Therefore, in this case, bacterial pyomyositis with paraspinal abscess was likely associated with the recent history of trauma, emphasized by the recent fractures, which is consistent with previously reported cases.

## Conclusions

Infectious pyomyositis is an uncommon bacterial infection of the striated muscle that can affect adults. Nonspecific symptoms and delayed diagnosis are the main problems with this disease. Diagnostic suspicion is critical, and imaging methods can help confirm the diagnosis. This case highlights the importance of a detailed clinical examination and of employing a multimodal imaging approach, which has turned it imperative to obtain a diagnosis in pathologies with nonspecific clinical manifestations, such as infectious pyomyositis, particularly in patients without evident risk factors.
